# Concurrent Ovarian and Tubal Ectopic Pregnancy After IVF-ET: Case Report and Literature Review

**DOI:** 10.3389/fphys.2022.850180

**Published:** 2022-04-04

**Authors:** Yating Huang, Qin Huang, Jinglan Liu, Mengxi Guo, Yuan Liu, Dongmei Lai

**Affiliations:** ^1^ School of Medicine, The International Peace Maternity and Child Health Hospital, Shanghai Jiaotong University, Shanghai, China; ^2^ Shanghai Key Laboratory of Embryo Original Disease, School of Medicine, Shanghai Jiaotong University, Shanghai, China

**Keywords:** ovarian pregnancy, tubal ectopic pregnancy, *in vitro* fertilization and embryo transfer, laparoscopy, mutiple embryo transfer

## Abstract

Ovarian pregnancy (OP) coupled with tubal ectopic pregnancy is rare. We present a case of coexistent ovarian and tubal ectopic pregnancies in the same adnexa resulting from *in vitro* fertilization and embryo transfer (IVF-ET) for tubal occlusion. The patient presented with mild vaginal bleeding without abdominal pain. OP was diagnosed *via* sonographic findings of an ectopic gestational sac (GS) and yolk sac that seemed to be inside her left ovary. Laparoscopic exploration confirmed this diagnosis, and ipsilateral tubal ectopic pregnancy was suspected during surgery. The patient underwent left salpingectomy and resection of the ovarian lesion. A subsequent histopathological examination verified the diagnosis of coexistent ovarian and tubal ectopic pregnancy. Though the mechanism underlying concurrent OP and tubal ectopic pregnancy is still unclear, clinicians should be cautious of potential combined ectopic pregnancy when dealing with patients who have received more than one embryo transfer.

## Introduction

Ovarian pregnancy (OP), a rare subgroup of ectopic pregnancy, comprised 0.15–3.2% of ectopic pregnancies (Bouyer et al., 2002; Raziel et al., 2004; Choi et al., 2011). It is even rarer for it to co-occur with tubal ectopic pregnancy (TP). To the best of our knowledge, only a few such cases have been reported (M Sueldo et al., 2014; Eom et al., 2018; Trindade et al., 2019).

Overall, the risk factors for OP are similar to those of TP, including a history of pelvic inflammatory disease, IVF, and previous abdominal surgery (Kamath et al., 2010; Weiss et al., 2016; Jennings and Krywko, 2020). In addition, polycystic ovarian syndrome, intra-uterine device usage, and endometriosis are also considered specific risk factors for OP patients (Wang et al., 2013; Parker and Srinivas, 2016; Alalade et al., 2017).

Most OP patients present with non-specific symptoms with lower abdominal pain and/or mild vaginal bleeding (Choi et al., 2011; Parker and Srinivas, 2016). If ultrasound fails to detect any signs of combined pregnancy, an integral preoperative diagnosis including OP can be difficult to determine. Most cases have been confirmed by operation and postoperative pathological analysis. Currently, the diagnosis of OP is still based on the original criteria reported by (Spiegelberg, 1878).

Here, we report a case of coexistent OP with unexpected TP after the transfer of two fresh embryos. Accordingly, we review several previous works for clinical features and advances in diagnosis and treatment.

## Case Report

A 35-year-old nulligravid woman was hospitalized with a suspected OP 28 days after the transfer of two fresh embryos. Her previous menstrual cycles had been irregular, with a period occurring every one to 3 months that lasted three to 5 days, with average flow and mild dysmenorrhea. She had experienced a hysterography (HSG), which revealed a complete obstruction in the right fallopian tube and a partial obstruction in the left fallopian tube. She underwent two cycles of conventional IVF, both of which failed. A third IVF procedure was performed. Ovarian stimulation was performed with clomiphene citrate 100 mg (days 3–7), followed by daily injections of HMG 75 IU/150 IU based on follicular response. When the follicle was found to have reached a size of ≥16 mm, GnRH antagonist Cetrorelix 0.25 mg was administered. Then, five eggs were retrieved, and, under ultrasonographic guidance, two fresh embryos (one 9-celled embryo/grade II and one 12-celled embryo/grade II) were transferred to cleavage state (D3). Dydrogesterone (30 mg/day, orally; Duphaston^®^, Abbott Biologicals B.V., Netherlands) was prescribed for luteal support. Two weeks after transfer, the patient was confirmed to have conceived, and the human chorionic gonadotrophin and beta fraction (β-hCG) levels were 414.2 IU/L. About 3 weeks after transfer, she had slight vaginal bleeding for 1 day, but no other discomfort.

**FIGURE 1:**
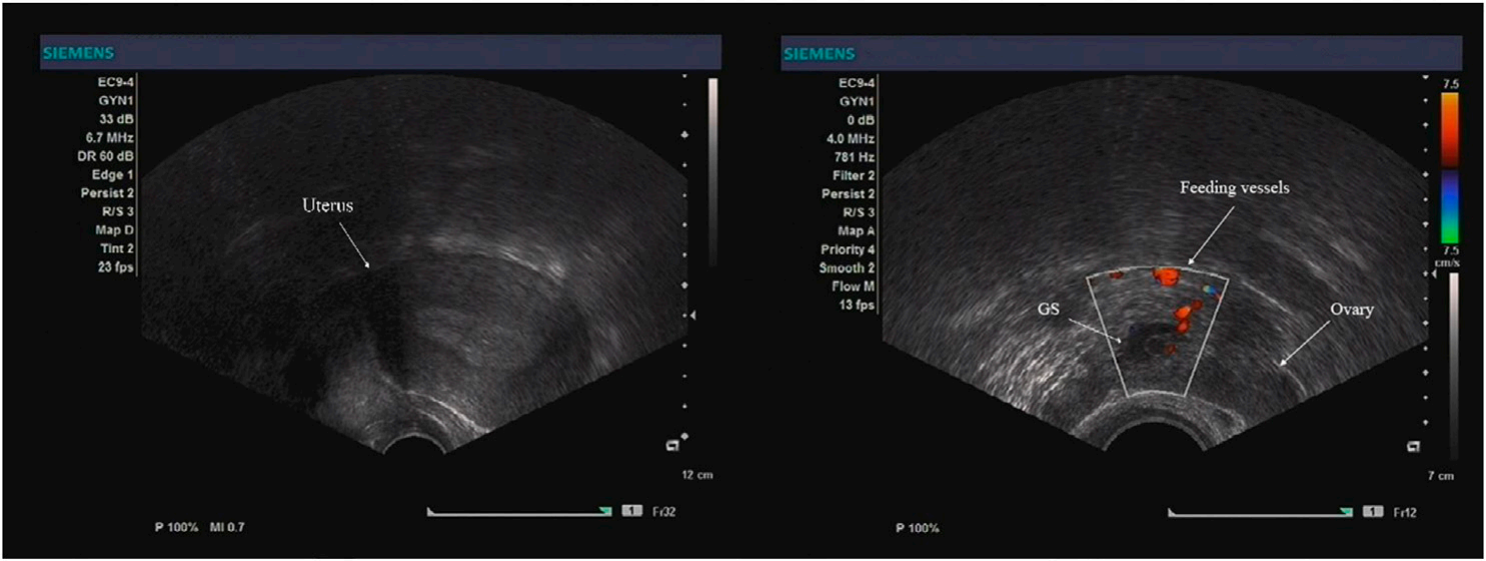
Ultrasound image of the left ovarian ectopic pregnancy, showing the GS with a yolk sac inside and feeding vessels around.

Routine viability ultrasonography was performed at 4-week gestation. Transvaginal ultrasonography revealed an empty uterus measuring 71 mm × 65 mm × 54 mm with an endometrial thickness of 12 mm. Her right ovary and tubal structures seemed to be normal, and a 30 × 25 × 20 mm heterogeneous mass was noted in the left adnexal area. A gestational sac (GS) with a beating fetal heart was seen inside, surrounded by ovary-like tissue, suggesting OP. Vascular proliferation was detected around the GS under power Doppler (Figure 1).

The patient was asymptomatic and hemodynamically stable when sent to the wards. On bimanual examination, no tenderness or masses were palpable on any side of her abdomen; no cervical pain was reported. A speculum examination showed no active bleeding at the cervix and only a trace of bloodstain on the vaginal wall. Furthermore, no abnormality was found in laboratory analysis of blood routine and blood biochemistry. The patient denied any history of endometriosis, pelvic inflammatory disease, or other relevant medical history.

**FIGURE 2:**
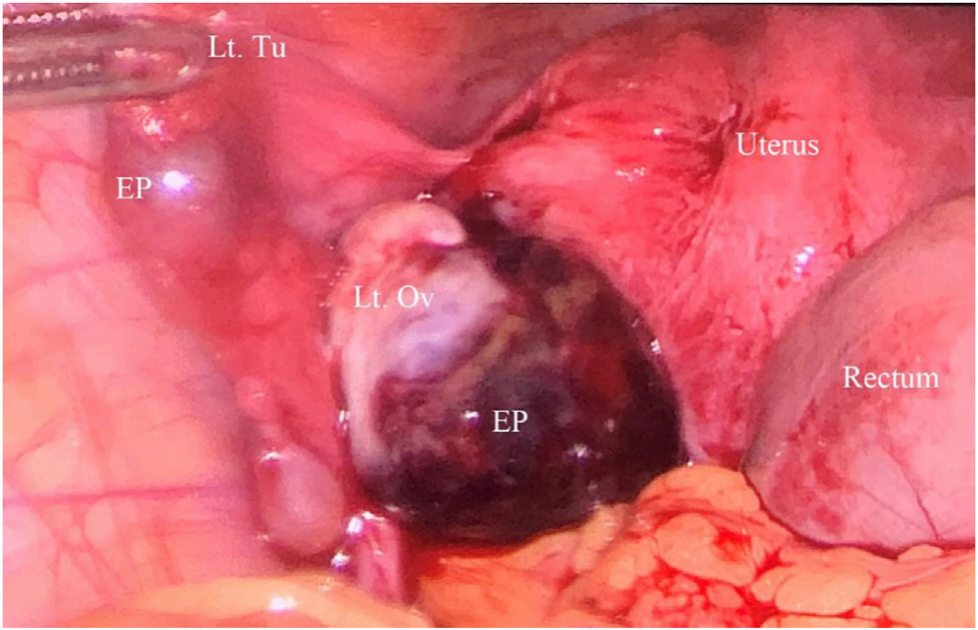
Laparoscopic view of unruptured left ovarian pregnancy and ipsilateral tubal pregnancy (Lt. Tu = Left Fallopian tube, Lt. Ov = Left Ovary, EP = ectopic pregnancy).

A provisional diagnosis of left OP was made, and laparoscopic exploration was performed immediately. The surgeons explored the pelvic and abdominal cavities after aspirating about 200 ml of blood from the pelvis. The right fallopian tube and ovary were found to be normal, and the left ovary was enlarged and blueish, swelling to 6 cm in diameter. The left tube was exposed in a routine manner and found to be slightly distended and purple in appearance in the ampulla, which was dilated about 1.5 cm in diameter; both were intact (Figure 2). Considering the patient’s recent embryo transfer, surgeons decided to perform the left salpingectomy and remove ectopic tissue while preserving the ovary. The trophoblastic tissue was removed from the left ovary with monopolar laparoscopic forceps, and the ovary was reconstructed with vicryl.

**FIGURE 3:**
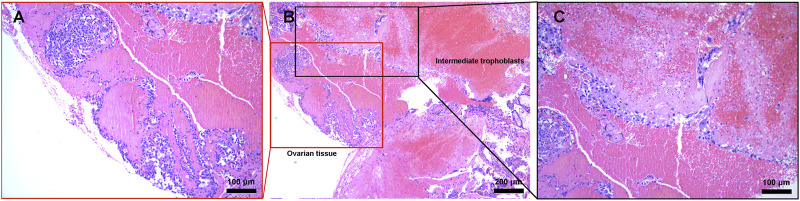
Histopathological image showed ovarian tissue and intermediate trophoblasts were seen in the pathology slide of ovarian lesion. Scale bars, **(A)**, 100 μm **(B)**, 200 μm and **(C)**, 100 μm.

**FIGURE 4:**
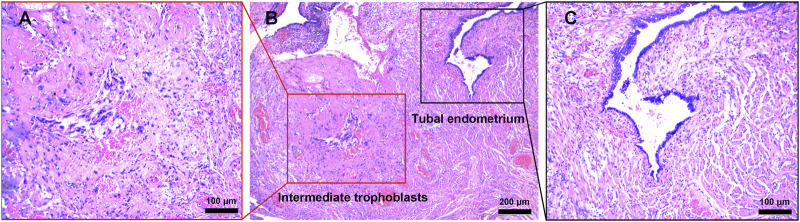
Histopathological staining showed a small amount of intermediate trophoblasts infiltration into the fallopian tube tissue. Scale bars, **(A)**, 100 μm **(B)**, 200 μm and **(C)**, 100 μm.

Pathological examination with hematoxylin and eosin staining of the surgical specimen showed a left OP (Figure 3) and ipsilateral tubal pregnancy (Figure 4) with the presence of trophoblastic tissues.

## Discussion

Combined pregnancy is rare and poses early diagnostic challenges. In existing reports, the clinical features of OP and TP patients have been unspecific, thus posing a dilemma for rupture and massive intra-abdominal bleeding with delayed diagnosis (Trindade et al., 2019). Particularly in cases of OP, pre-operative diagnosis is difficult to perform; however, this situation is improving owing to recent advances in ultrasound. Some authors state that the ultrasonic appearance suggestive of OP is a hypo-echoic, predominantly solid mass surrounded with blood flow signals (Comstock et al., 2005; Joseph and Irvine, 2012; Alalade et al., 2017), which is called the “ring of fire” structure. Moreover, an ectopic yolk sac and cardiac activity can facilitate provisional diagnosis of OP during ultrasonography (Comstock et al., 2005). It should be noted that advances in ultrasound technology can rectify the shortcomings of intra- and post-operative diagnosis involving the criteria established. MRI can also be an effective adjunct to ultrasound in the case of a patient with a hemodynamically stable status (Alalade et al., 2017; Ramanathan et al., 2018).

Here, we reported a case of concurrent OP and TP following IVF-ET to determine the causes thereof. ART was observed as a major risk factor in this case, as shown in Supplementary Table S1. This was consistent with three previous reports (M Sueldo et al., 2014; Eom et al., 2018; Trindade et al., 2019). Among these, M Sueldo et al. and Trindade et al. reported concurrent OP and TP after the transfer of two fresh embryos, and Eom et al. reported a patient who had undergone IUI treatment. Importantly, multiple embryo transfer was believed to be an important cause that significantly raised the rate of ectopic pregnancy over elective single transfer (Clayton et al., 2006; Bu et al., 2016). Several retrospective cohort studies have shown that more patients following IVF were found to be associated with fresh embryo transfer than frozen embryo transfer (FET) (Ishihara et al., 2011; Shapiro et al., 2011; Shapiro et al., 2012; Huang et al., 2014; Fang et al., 2015; Londra et al., 2015). In addition, receiving an embryo at the cleavage state (D3) was associated with a higher risk of ectopic pregnancy than a blastocyst on day 5 (Huang et al., 2014; Fang et al., 2015). Thus, fresh embryo transfer at the cleavage stage and multiple embryo transfer may be risk factors for multi-site ectopic pregnancy after ART. Other specific risk factors were also speculated; moreover, a high volume of culture medium was used when loading embryo or embryos, when there was an excessive ovarian response, in the transfer of an embryo in an abnormally high estrogen environment, and when there was a decreased transfer distance from the fundus (Pope et al., 2004; Chang and Suh, 2010; Wang et al., 2013; Jeon et al., 2016; Weiss et al., 2016; Lin et al., 2019).

Two hypotheses may explain the mechanism underlying concurrent ectopic pregnancy. First, the embryo or blastocyst may migrate in retrograde through the tube and implant in the ovary. Second, it may pass into one of the puncture sites created by the aspiration needle (Boronow et al., 1965). During the fresh cycle, ovarian injury after oocyte retrieval may provide an opportunity for ectopic implantation (Ishihara et al., 2011). Elevation of the E2/P ratio with the administration of stimulating drugs or exogenous hormone supplementation may lead to uncoordinated movement of the uterus and fallopian tubes, causing the embryo to migrate in reverse into the abdominal cavity (Wang et al., 2013; Fang et al., 2015). Another mechanism is some manner of interference in the release of the ovum from the follicle, followed by fertilization *in situ* by the sperm (Dolinko et al., 2018).

As with tubal pregnancies, surgery remains the first choice treatment (Dolinko et al., 2018), especially for patients with significant hypoxia or hemodynamic instability (Odejinmi et al., 2011). Furthermore, minimal access surgery is now becoming a universal option (Joseph and Irvine, 2012). Although wedge resection of the ovary is still the most common procedure for OP (Choi et al., 2011), enucleation of the gestational product is receiving increasing acceptance from doctors, as it is considered the gentlest type of operation, able to preserve as much ovarian cortex as possible (Alkatout et al., 2011). Such a procedure includes enucleating the GS from the ovary, bluntly or with the help of monopolar or bipolar cautery (Einenkel et al., 2000; Nadarajah et al., 2002; Andrade et al., 2015), and subsequently hemostasis with electrocoagulation, thereby protecting the ovarian function to the greatest extent possible. However, for patients in life-threatening situations (e.g., excessive bleeding, difficult hemostasis), it may be appropriate to remove the entire ovary.

Furthermore, methotrexate therapy, including systemic application and local intra-GS injection (Shamma and Schwartz, 1992; Mittal et al., 2003; Dolinko et al., 2018), could be considered an alternative treatment with strict indications and monitoring (Andrade et al., 2015). However, it is not recommended as a first-line treatment by the American Society of Reproductive Medicine (ASRM).

Co-existing ectopic pregnancies may be misdiagnosed and treatment may be delayed, which may lead to life-threatening complications and necessitate additional surgery. Upon review of reported cases, we developed several specifications for the prevention of co-existing ectopic pregnancy after IVF-ET: 1) clinicians should be alert that more than one embryo was transferred in IVF-ET, or ovarian hyperstimulation was conducted in the pregnancy; 2) clinicians should be alert to abnormal changes in β-HCG after IVF-ET; 3) ultrasonography may show an empty uterus with GS occupying the position of the adnexa; 4) because either ipsilateral or contralateral ovarian and tubal pregnancy could occur, laparoscopic exploration of both lateral fallopian tubes and ovaries is needed, and clinicians should pay attention to laparoscopic images showing purple bulging of the tube or ovarian hemorrhage; and 5) pathologic evidence may include ovarian tissue in the wall of the GS and a GS in the fallopian tubal tissue.

## Conclusion

Concurrent OP and tubal pregnancy after ART have been reported in a few cases. In this report, we found that preoperative diagnosis involves considerable challenges. Risk factors include the transfer of multiple embryos in IVF-ET or ovarian hyperstimulation. As such, surgery remains the preferred treatment. Routine intra-operatory inspection of both fallopian tubes and ovaries is strongly recommended in any ectopic pregnancy, especially in high-risk patients.

## Data Availability

The original contributions presented in the study are included in the article/Supplementary Material, further inquiries can be directed to the corresponding author.
